# Evaluation of Optimal Diastolic Blood Pressure Range Among Adults With Treated Systolic Blood Pressure Less Than 130 mm Hg

**DOI:** 10.1001/jamanetworkopen.2020.37554

**Published:** 2021-02-17

**Authors:** Jingen Li, Virend K. Somers, Xiang Gao, Zhuo Chen, Jianqing Ju, Qian Lin, Essa A. Mohamed, Shahid Karim, Hao Xu, Lijing Zhang

**Affiliations:** 1 Department of Cardiology, Dongzhimen Hospital, Beijing University of Chinese Medicine, Beijing, China; 2 Department of Cardiovascular Medicine, Mayo Clinic, Rochester, Minnesota; 3 Internal Medicine Division, Tieying Hospital of Fengtai District, Beijing, China; 4 Cardiovascular Diseases Center, Xiyuan Hospital, China Academy of Chinese Medical Sciences, National Clinical Research Center for Chinese Medicine Cardiology, Beijing, China

## Abstract

**Importance:**

Extremely low diastolic blood pressure has been reported to be associated with increased adverse cardiovascular events (ie, the diastolic J-shape phenomenon); however, current US guidelines recommend an intensive blood pressure target of less than 130/80 mm Hg without mentioning the lower limits of diastolic blood pressure.

**Objectives:**

To evaluate whether there is a diastolic J-shape phenomenon for patients with an treated systolic blood pressure of less than 130 mm Hg and to explore the safe and optimal diastolic blood pressure ranges for this patient population.

**Design, Setting, and Participants:**

This cohort study analyzed outcome data of patients at high cardiovascular risk who were randomized to intensive or standard blood pressure control and achieved treated systolic blood pressure of less than 130 mm Hg in the Systolic Blood Pressure Intervention Trial (SPRINT) and Action to Control Cardiovascular Risk in Diabetes–Blood Pressure (ACCORD-BP) trial. Data were collected from October 2010 to August 2015 (SPRINT) and from September 1999 to June 2009 (ACCORD-BP). Data were analyzed from January to May 2020.

**Exposure:**

Treated diastolic blood pressure, divided in intervals of less than 60, 60 to less than 70, 70 to less than 80, and 80 mm Hg and greater.

**Main Outcomes and Measures:**

The primary outcome was a composite of all-cause death, nonfatal myocardial infarction, and nonfatal stroke. A composite cardiovascular outcome, including cardiovascular death, nonfatal myocardial infarction, and nonfatal stroke, was among the key secondary outcomes.

**Results:**

A total of 7515 patients (mean [SD] age, 65.6 [8.7] years; 4553 [60.6%] men) were included in this analysis. The nominally lowest risk was observed at a diastolic blood pressure between 70 and 80 mm Hg for the primary outcome, the composite cardiovascular outcome, nonfatal myocardial infarction, and cardiovascular death. A mean diastolic blood pressure of less than 60 mm Hg was associated with significantly increased risk of the primary outcome (hazard ratio [HR], 1.46; 95% CI, 1.13-1.90; *P* = .004), the composite cardiovascular outcome (HR, 1.74; 95% CI, 1.26-2.41; *P* = .001), nonfatal myocardial infarction (HR, 1.73; 95% CI, 1.15-2.59; *P* = .008), and nonfatal stroke (HR, 2.67; 95% CI, 1.26-5.63; *P* = .01).

**Conclusions and Relevance:**

This cohort study found that lowering diastolic blood pressure to less than 60 mm Hg was associated with increased risk of cardiovascular events in patients with high cardiovascular risk and an treated systolic blood pressure less than 130 mm Hg. The finding that a diastolic blood pressure value between 70 and 80 mm Hg was an optimum target for this patient population merits further study.

## Key Points

**Question:**

What are the safe and optimal diastolic blood pressure (DBP) ranges among adults with treated systolic blood pressure (SBP) of less than 130 mm Hg?

**Findings:**

In this cohort study of 7515 patients from 2 randomized clinical trials who had treated SBP of less than 130 mm Hg, a DBP of less than 60 mm Hg was associated with increased cardiovascular risk and a DBP between 70 and 80 mm Hg was associated with lower cardiovascular risk.

**Meaning:**

The findings suggest that a DBP of less than 60 mm Hg may be harmful and a DBP between 70 and 80 mm Hg is an optimal target for patients with treated SBP of less than 130 mm Hg; this topic merits further study.

## Teaser

This cohort study evaluates whether diastolic blood pressure (BP) among adults with treated systolic BP of less than 130 mm Hg is associated with all-cause mortality, nonfatal myocardial infarction, and nonfatal stroke.

## Introduction

High blood pressure (BP) is among the most important modifiable risk factors for cardiovascular disease and death.^1,2^ Prevailing concepts regarding BP have changed dramatically over time. Results of the Framingham Heart Study have shifted the emphasis on diastolic BP (DBP) to systolic BP (SBP) by showing that SBP was a more important risk factor for cardiovascular outcomes.^2^ This has generated debate and relative neglect of the role of DBP in cardiovascular risk estimation.^3^ Whether there is a diastolic J-shape phenomenon, meaning that both high and low DBP could increase cardiovascular risk, is among the most controversial issues.

Debate on the diastolic J-shape phenomenon originated from an observation of low DBP and increased risk of death from myocardial infarction.^4^ Although there are studies showing linear associations between DBP and cardiovascular risk,^5,6^ more studies have reported a diastolic J-shape phenomenon.^7,8,9,10,11,12,13,14,15^ It seems certain that extremely low DBP is harmful. However, mainly driven by the Systolic Blood Pressure Intervention Trial (SPRINT) trial,^16^ which showed improved prognosis with intensive SBP lowering, the 2017 American Heart Association (AHA) Hypertension Guidelines^17^ recommend an intensive BP target of less than 130/80 mm Hg, with no recommendation on lower limits of DBP. Intensive SBP lowering could result in an excessive reduction of DBP, generating concern that the therapeutic harm-benefit equation will shift to harm considering the potential diastolic J-shape phenomenon. To avoid potential harm, the 2018 European Society of Cardiology (ESC) Hypertension Guidelines have recommended an optimal DBP target of between 70 and 80 mm Hg for patients with all risk levels.^18^ Given the great influence of the 2017 AHA guideline and the argument that the diastolic J-shape phenomenon only exists in the presence of high SBP (ie, >140 mm Hg),^19^ it is critical to know whether the diastolic J-shape phenomenon exists in patients with SBP of less than 130 mm Hg and, if it does exist, what the safe and optimal DBP ranges are for this population.

The SPRINT^16^ and the Action to Control Cardiovascular Risk in Diabetes–Blood Pressure (ACCORD-BP)^20^ trials randomized 14 094 patients to intensive BP control (ie, SBP <120 mm Hg) and standard BP control (ie, SBP <140 mm Hg). The objectives of the present study were, first, to determine whether a diastolic J-shape phenomenon was evident with treated SBP of less than 130 mm Hg and, second, to investigate the safe and optimal DBP ranges in the presence of a guideline-recommended SBP of less than 130 mm Hg, using outcome data from SPRINT and ACCORD-BP.

## Methods

### Design and Participants

This cohort study was reported in adherence to the Strengthening the Reporting of Observational Studies in Epidemiology (STROBE) reporting guideline. The designs and outcomes of SPRINT^16^ and ACCORD-BP^20^ have been reported previously, and data were acquired from the National Heart, Lung, and Blood Institute BioLINCC Repository.^21^ In brief, SPRINT was a randomized, single-masked trial with 9361 patients at high cardiovascular risk randomized to an SBP target of either less than 140 mm Hg (the standard treatment group) or less than 120 mm Hg (the intensive treatment group) for a median of 3.26 years. Patients enrolled were aged 50 years or older with an SBP of 130 to 180 mm Hg with increased risk of cardiovascular disease but without diabetes or a history of stroke. Increased cardiovascular risk was defined by 1 or more of the following: clinical or subclinical cardiovascular disease other than stroke; chronic kidney disease, excluding polycystic kidney disease, with an estimated glomerular filtration rate (eGFR) between 20 and less than 60 mL/min/1.73 m^2^ of body surface area, as calculated by the 4 variable Modification of Diet in Renal Disease equation; a 10-year risk of cardiovascular disease of 15% or greater on the basis of the Framingham risk score; or age 75 years or older.^16^ In the ACCORD-BP trial, 4733 patients were randomly assigned to either intensive (ie, SBP <120 mm Hg) or standard (ie, SBP <140 mm Hg) BP control for a median of 4.7 years. Patients enrolled were aged 40 to 79 years with type 2 diabetes and hemoglobin A_1c_ concentrations of 7.5% or more (to convert to proportion of total hemoglobin, multiply by 0.01) with history of cardiovascular disease or individuals aged 55 to 79 years with anatomical evidence of significant atherosclerosis, albuminuria, left ventricular hypertrophy, or at least 2 risk factors for cardiovascular disease.^20^ In SPRINT, targeting a SBP of less 120 mm Hg vs less than 140 mm Hg resulted in 25% reduction of adverse cardiovascular events.^16^ The results of ACCORD-BP showed no difference in the composite cardiovascular outcome but did find a decrease of stroke.^20^ The studies were approved by the institutional review board at each participating site, and written informed consent was obtained from all participants. The present study protocol was approved by medical ethics committee of Xiyuan Hospital, China Academy of Chinese Medical Sciences. For this post hoc analysis, patients who did not have any recorded BP after randomization or had missing key covariates were excluded.

### BP Measurement

In the SPRINT^16^ and ACCORD-BP^20^ trials, BP was measured seated using a similar oscillometric device (Model 907XL in SPRINT^22^ and Model 907 in ACCORD-BP,^20^ Omron Healthcare). A mean of 3 subsequent BP measurements was recorded as baseline and follow-up BP. BPs were measured after 5 minutes of rest in both SPRINT and ACCORD-BP.^23^ BP measurements were scheduled at baseline, monthly for the first 3 months, and every 3 months thereafter in SPRINT. In the intensive group in ACCORD-BP, BP measurements were scheduled at baseline, every month for the first 4 months, and every 2 months thereafter; in the standard treatment group, BP measurements were scheduled at baseline, month 1, month 4, and every 4 months thereafter. For the present analysis, the treated SBP, DBP, and pulse pressure (PP) for each patient were computed by taking the mean of their BP measurement from month 3 to the last reading.

### Study Outcomes

The primary outcome of this analysis was a composite of all-cause death, nonfatal myocardial infarction, and nonfatal stroke. Secondary outcomes included the individual components of the primary outcome, cardiovascular death, and a composite cardiovascular outcome including cardiovascular death, nonfatal myocardial infarction, and nonfatal stroke.

### Statistical Analysis

Due to the similarity of the study design and study population, we combined data from the SPRINT and ACCORD-BP trials. To avoid potential bias by combining data from 2 different studies, we also analyzed data from the 2 trials separately. Patients with achieved SBP of less than 130 mm Hg were selected and divided into subgroups on the basis of their mean achieved DBP at the following cutoffs: less than 60, 60 to less than 70, 70 to less than 80, and at least 80 mm Hg, with 70 to less than 80 mm Hg as the reference (ie, hazard ratio [HR] considered 1). Groups were tested for differences using 1-way analysis of variance for continuous data and the χ^2^ test for categorical data. HRs for all outcomes were analyzed by DBP category and tested for differences using Cox regression, adjusting for study, study group (intensive vs standard BP control; intensive vs standard glycemic control), and baseline patient characteristics (ie, age, sex, tobacco use, education, heart rate, body mass index [BMI, calculated as weight in kilograms divided by height in meters squared], renal function, serum level of low density lipoprotein cholesterol [LDL-C] and glucose, history of hypertension, history of cardiovascular disease, family history of cardiovascular disease, and concomitant medications). History of cardiovascular disease was defined as history of myocardial infarction, stroke, acute coronary syndrome, coronary artery bypass grafting or percutaneous coronary intervention, other revascularization (ie, carotid, peripheral, abdominal aortic aneurysm repair), left ventricular hypertrophy, ankle brachial index of 0.9 or less, and at least 50% stenosis of artery. The association between mean achieved DBP and DBP changes from baseline as continuous variables with SBP of less than 130 mm Hg was also analyzed with restricted cubic splines that allow exploration of nonlinear associations.^24^ Likelihood-ratio test was used to assess whether the association was really nonlinear. To avoid potential reverse associations, we excluded patients with any primary or secondary events within 30 days after randomization. In addition, sensitivity analyses were conducted by excluding patients with mean achieved SBP of less than 110 mm Hg, by additional adjustment of baseline PP as a categorical variable (≥60 mm Hg or not) and baseline DBP in the Cox regression model, and by additional adjustment of baseline PP (≥60 mm Hg or not), baseline SBP, and achieved SBP in the Cox regression model. We also analyzed the association of intensive vs standard BP control with cardiovascular and mortality outcomes in a subgroup of patients with baseline DBP of less than 60 mm Hg in the ACCORD-BP and SPRINT trials separately. All analyses were done with SPSS statistical software version 20.0 (IBM Corp) and RStudio version 1.2.5033 (survival and rms packages; R Project for Statistical Computing). A 2-tailed *P* < .05 was considered statistically significant.

## Results

### Baseline Characteristics

**Figure 1.**
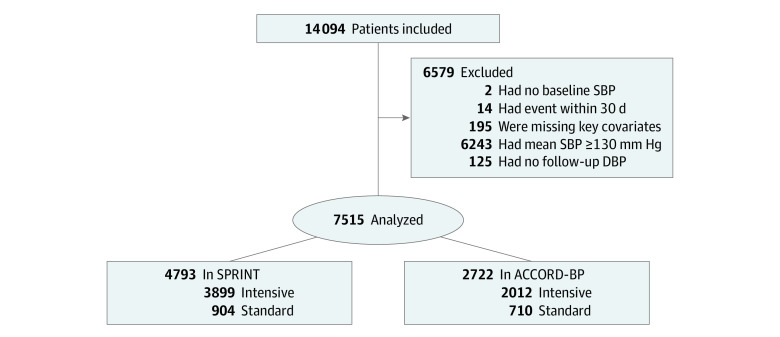
Patient Selection and Allocation Chart ACCORD-BP indicates Action to Control Cardiovascular Risk in Diabetes–Blood Pressure; DBP, diastolic blood pressure; SBP, systolic blood pressure; and SPRINT, Systolic Blood Pressure Intervention Trial.

In SPRINT and ACCORD-BP trials, we included 7515 patients (mean [SD] age 65.6 [8.7] years) with 4553 (60.6%) men (Figure 1). Most patients achieved a DBP of between 60 and less than 70 mm Hg (3250 [43.2%]) and between 70 and less than 80 mm Hg (2545 [33.9%]) (Table). Patients who achieved lower DBP were generally older (eg, 60 to <70 mm Hg vs ≥80 mm Hg: mean [SD] age, 66.9 [8.2] years vs 59.0 [5.6] years; *P* < .001), had lower mean (SD) BMI (eg, 60 to <70 mm Hg vs ≥80 mm Hg: 30.9 [5.7] vs 31.0 [5.9]; *P* < .001), had lower mean (SD) baseline DBP (eg, 60 to <70 mm Hg vs ≥80 mm Hg: 75.0 [9.0] vs 89.0 [10.3]; *P* < .001), had higher mean (SD) baseline SBP (eg, 60 to <70 mm Hg vs ≥80 mm Hg: 136.8 [15.1] vs 135.0 [15.1]; *P* = .004), had lower mean (SD) LDL-C levels (eg, 60 to <70 mm Hg vs ≥80 mm Hg: 108.0 [34.9] mg/dL vs 120.7 [38.4] mg/dL [to convert to millimoles per liter, multiply by 0.0259]; *P* < .001), had higher mean (SD) glycemic levels (eg, 60 to <70 mm Hg vs ≥80 mm Hg: 129.0 [51.2] mg/dL vs 111.0 [38.4] mg/dL [to convert to millimoles per liter, multiply by 0.0555]; *P* < .001), were less likely to currently use tobacco (eg, 60 to <70 mm Hg vs ≥80 mm Hg: 369 [11.4%] vs 121 [25.4%]; *P* < .001), and more likely to have a history of cardiovascular disease (1509 [46.4%] vs 114 [23.9%]; *P* < .001). Full clinical and demographic characteristics grouped by treated DBP are presented in the Table.

**Table.** Demographic Characteristics and Baseline Conditions by Mean Achieved DBP at Mean Achieved SBP of Less Than 130 mm Hg

Characteristic	Patients with mean achieved DBP, No. (%)				*P* value
	<60 mm Hg	60 to <70 mm Hg	70 to <80 mm Hg	≥80 mm Hg	
Patients	1244 (16.6)	3250 (43.2)	2545 (33.9)	476 (6.3)	NA
Achieved SBP, mean (SD), mm Hg	119.1 (6.1)	119.3 (5.6)	121.1 (5.3)	124.7 (4.3)	<.001
Baseline					
SBP, mean (SD), mm Hg	137.4 (15.1)	136.8 (15.1)	136.0 (14.9)	135.0 (15.1)	.004
DBP, mean (SD), mm Hg	65.7 (8.6)	75.0 (9.0)	82.6 (9.4)	89.0 (10.3)	<.001
PP ≥60	1011 (81.3)	1658 (51.0)	599 (23.5)	31 (6.5)	<.001
Heart rate, mean (SD), bpm	65.0 (11.4)	68.4 (11.67)	70.8 (12.1)	73.8 (12.4)	<.001
Age, y					
Mean (SD)	71.9 (8.3)	66.9 (8.2)	62.2 (7.5)	59.0 (5.6)	<.001
<65	287 (23.1)	1456 (44.8)	1743 (68.5)	404 (84.9)	
65 to <75	437 (35.1)	1139 (35.0)	602 (23.7)	65 (13.7)	
≥75	520 (41.8)	655 (20.2)	200 (7.9)	7 (1.5)	
Women	450 (36.2)	1298 (39.9)	1027 (40.4)	187 (39.3)	.08
Race/ethnicity					
Hispanic	101 (12.9)	285 (13.3)	277 (14.5)	51 (12.4)	<.001
Black	121 (15.4)	447 (20.9)	597 (31.3)	182 (44.2)	
White	505 (64.3)	1222 (57.1)	916 (48.1)	166 (40.3)	
Other^a^	58 (15.5)	187 (50.1)	115 (30.8)	13 (3.5)	
BMI, mean (SD)	29.3 (5.4)	30.9 (5.7)	31.6 (6.0)	31.0 (5.9)	<.001
Obesity	490 (39.4)	1629 (50.1)	1426 (56.0)	313 (65.8)	<.001
Baseline eGFR MDRD, mean (SD), mL/min/1.73 m^2^	75.1 (24.3)	80.2 (28.3)	81.4 (25.1)	79.7 (26.4)	<.001
Fasting LDL cholesterol, mean (SD), mg/dL	103.5 (32.9)	108.0 (34.9)	115.9 (37.0)	120.7 (37.4)	<.001
Fasting glucose, mean (SD), mg/dL	129.1 (49.5)	129.0 (51.2)	123.1 (51.0)	111.0 (38.4)	<.001
Education					
<High school	161 (12.9)	352 (10.8)	226 (8.9)	41 (8.6)	.02
High school	241 (19.4)	661 (20.3)	518 (20.4)	91 (19.1)	
Some college or technical school	420 (33.8)	1128 (34.7)	879 (34.6)	171 (35.9)	
≥College graduate	422 (33.9)	1108 (34.1)	921 (36.2)	173 (36.3)	
Smoking status					
Current	100 (8.1)	369 (11.4)	422 (16.6)	121 (25.4)	<.001
Former	637 (51.3)	1445 (44.5)	965 (37.9)	174 (36.6)	
Never	505 (40.7)	1434 (44.2)	1157 (45.5)	181 (38.0)	
History					
Hypertension	1038 (83.4)	2709 (83.4)	2176 (85.5)	430 (90.3)	<.001
CVD^b^	681 (54.7)	1509 (46.4)	936 (36.8)	114 (23.9)	<.001
Family history of CVD^b^	717 (57.7)	1854 (57.0)	1478 (58.1)	276 (58.0)	.99
Current medication					
Aspirin	816 (65.7)	1796 (55.3)	1189 (46.8)	195 (41.1)	<.001
Statins	763 (61.7)	1815 (56.1)	1146 (45.2)	173 (36.6)	<.001
Study					
ACCORD-BP	530 (42.6)	1322 (40.7)	795 (31.2)	75 (15.8)	<.001
SPRINT	714 (57.4)	1928 (59.3)	1750 (68.8)	401 (84.2)	
Treatment group					
Intensive	1077 (86.6)	2689 (82.7)	1902 (74.7)	233 (48.9)	<.001
Standard	167 (13.4)	561 (17.3)	643 (25.3)	243 (51.1)	

Abbreviations: ACCORD-BP, Action to Control Cardiovascular Risk in Diabetes–Blood Pressure; BMI, body mass index (calculated as weight in kilograms divided by height in meters squared); bpm, beats per minute; CVD, cardiovascular disease; DBP, diastolic blood pressure; eGFR, estimated glomerular filtration rate; LDL, low-density lipoprotein; NA, not applicable; MDRD, modification of diet in renal disease; PP, pulse pressure; SBP, systolic blood pressure; SPRINT, Systolic Blood Pressure Intervention trial.

SI conversion factor: To convert LDL cholesterol to millimoles per liter, multiply by 0.0259; glucose to millimoles per liter, multiply by 0.0555.

^a^ Other races included Asian, American Indian, Native Hawaiian, and others.

^b^ CVD was defined as history of myocardial infarction, stroke, acute coronary syndrome, coronary artery bypass graph or percutaneous coronary intervention, other revascularization (ie, carotid, peripheral, abdominal aortic aneurysm repair), left ventricular hypertrophy, ankle brachial index of 0.9, or less and at least 50% stenosis of artery.

### Treated DBP and Outcomes

**Figure 2.**
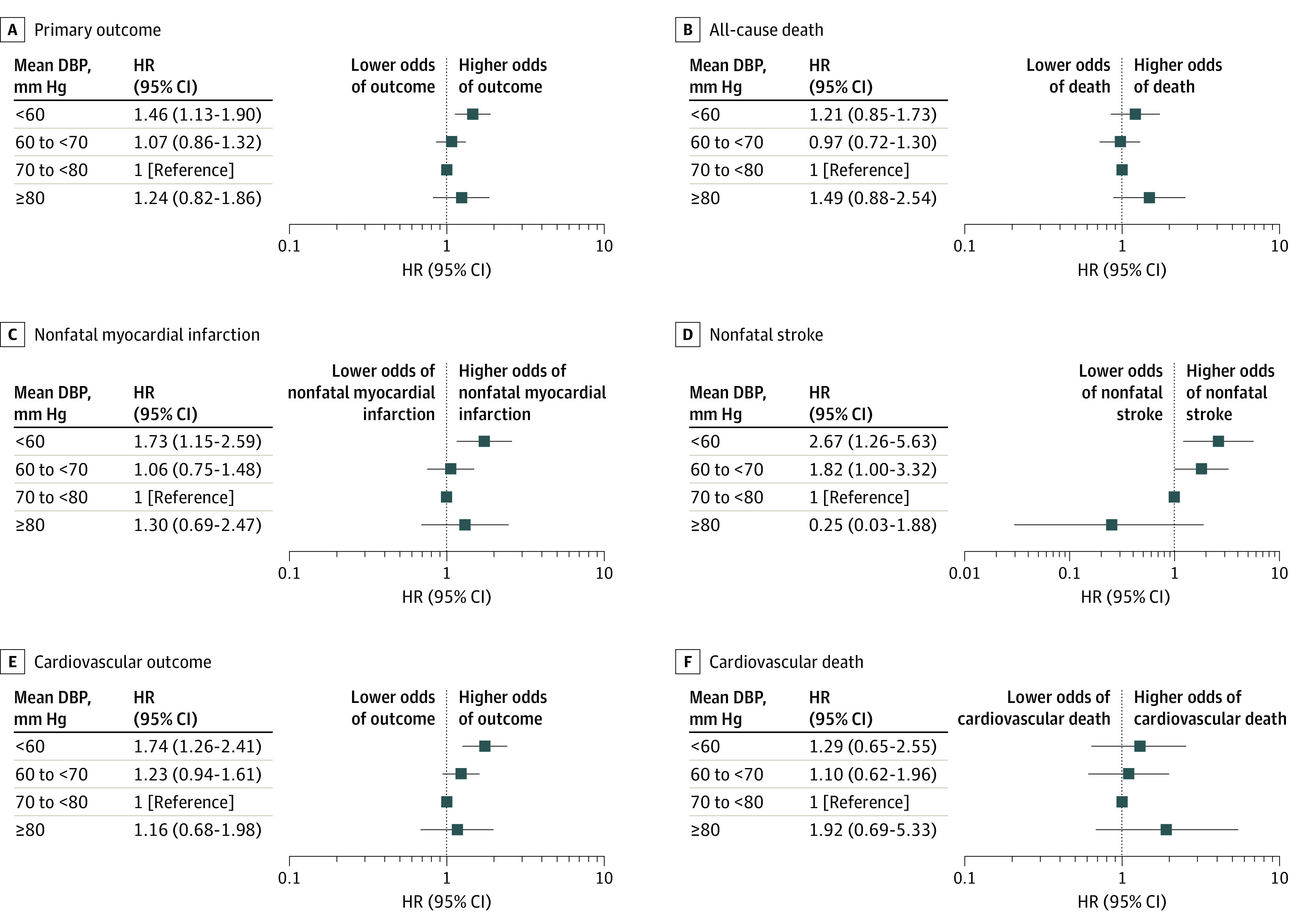
Adjusted Hazard Ratios (HRs) for Achieved Diastolic Blood Pressure (DBP) and Outcomes at Achieved Systolic BP of Less Than 130 mm Hg The primary outcome consisted of all-cause death, nonfatal myocardial infarction, and nonfatal stroke. The cardiovascular outcome was composed of cardiovascular death, nonfatal myocardial infarction, and nonfatal stroke.

The adjusted HRs for primary and secondary outcomes in different DBP groups are presented in Figure 2. (Event rates appear in eTable 1 in the Supplement.) The nominally lowest risk for all outcomes except all-cause death and nonfatal stroke was observed at a DBP level of 70 to less than 80 mm Hg. A significant increase of risk of the primary outcome (HR, 1.46; 95% CI, 1.13-1.90; *P* = .004), cardiovascular outcome (HR, 1.74; 95% CI, 1.26-2.41; *P* = .001), nonfatal myocardial infarction (HR, 1.73; 95% CI, 1.15-2.59; *P* = .008), and nonfatal stroke (HR, 2.67; 95% CI, 1.26-5.63; *P* = .01) was observed at DBP of less than 60 mm Hg. No significant associations were observed between achieved DBP of 80 mm Hg or greater and the primary outcome (HR, 1.24; 95% CI, 0.82-1.86; *P* = .30), all-cause death (HR, 1.49; 95% CI, 0.88-2.54; *P* = .14), nonfatal myocardial infarction (HR, 1.30; 95% CI, 0.69-2.47; *P* = .42), cardiovascular outcome (HR, 1.16; 95% CI, 0.68-1.98; *P* = .58), and cardiovascular death (HR, 1.92; 95% CI, 0.69-5.33; *P* = .21).

To test the robustness of our results, we conducted sensitivity analyses. First, with analysis of data from SPRINT and ACCORD-BP trials separately, similar results were observed (eFigure 1, eFigure 2, and eTable 2 in the Supplement). Second, after excluding patients who achieved an SBP of less than 110 mm Hg, we observed similar results as in main analyses (eFigure 3 in the Supplement). Finally, sensitivity analyses by additional adjustment of baseline PP and baseline DBP (eFigure 4 in the Supplement) and by additional adjustment of baseline PP, baseline SBP, and achieved SBP (eFigure 5 in the Supplement) also generated similar results.

The analysis of the association of intensive BP lowering in the subgroup with a baseline DBP of less than 60 mm Hg showed a nonsignificant increase of mortality risk by intensive BP lowering in both SPRINT (all-cause death: HR, 1.25; 95% CI, 0.65-2.34; cardiovascular death: HR, 1.69; 95% CI, 0.28-10.12) (eTable 3 in the Supplement) and ACCORD-BP (all-cause death: HR, 2.00; 95% CI, 1.001-4.00; cardiovascular death: HR, 1.25; 95% CI, 0.49-3.16 (eTable 4 in the Supplement), and no interaction between intensive BP treatment and baseline DBP was observed (eTable 3 and eTable 4 in the Supplement).

**Figure 3.**
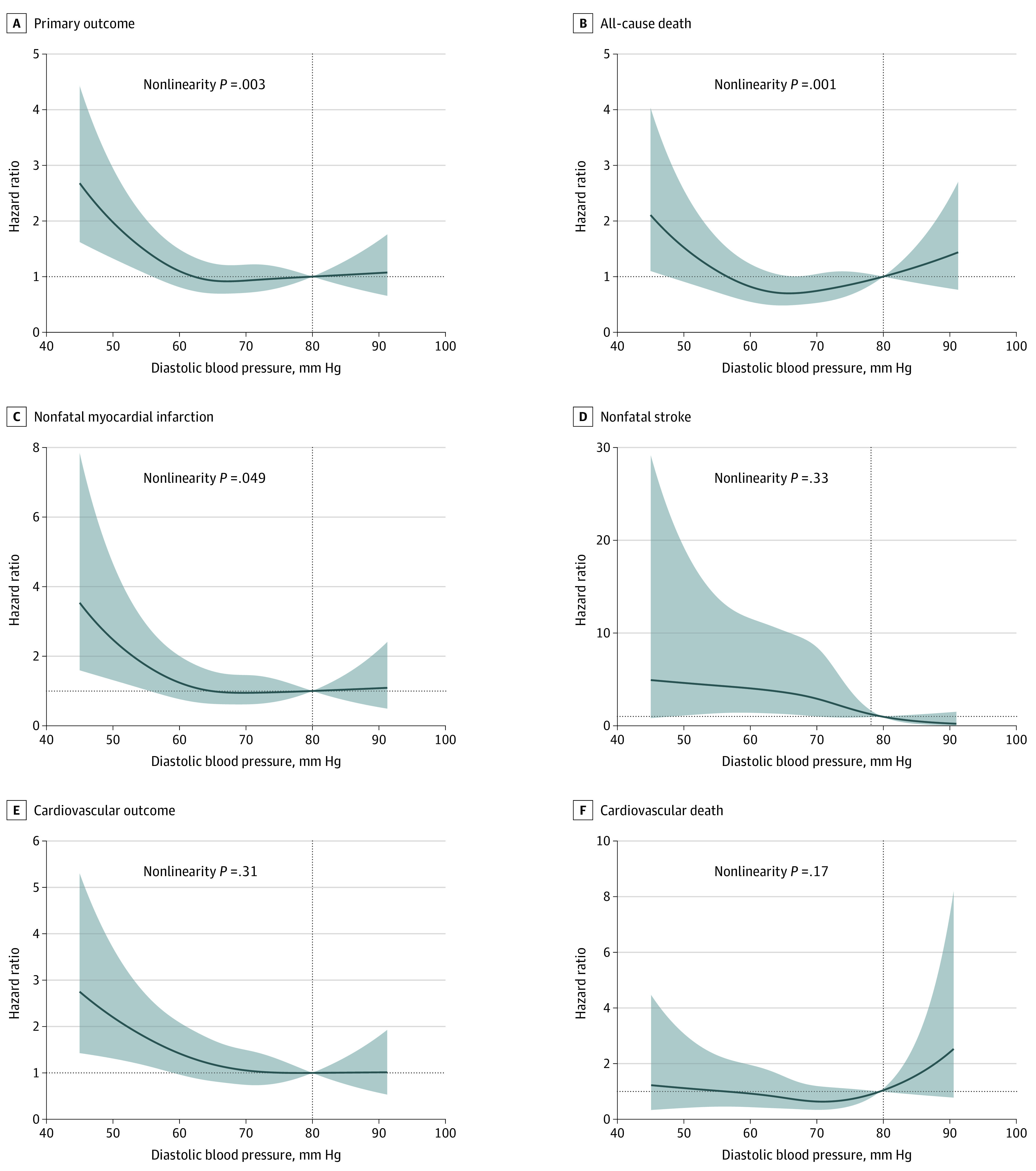
Cubic Splines of the Adjusted Hazard Ratios for Achieved Diastolic Blood Pressure The primary outcome consisted of all-cause death, nonfatal myocardial infarction, and nonfatal stroke. The cardiovascular outcome was composed of cardiovascular death, nonfatal myocardial infarction, and nonfatal stroke. The reference is diastolic blood pressure of 80 mm Hg. Shaded areas indicate 95% CIs.

**Figure 4.**
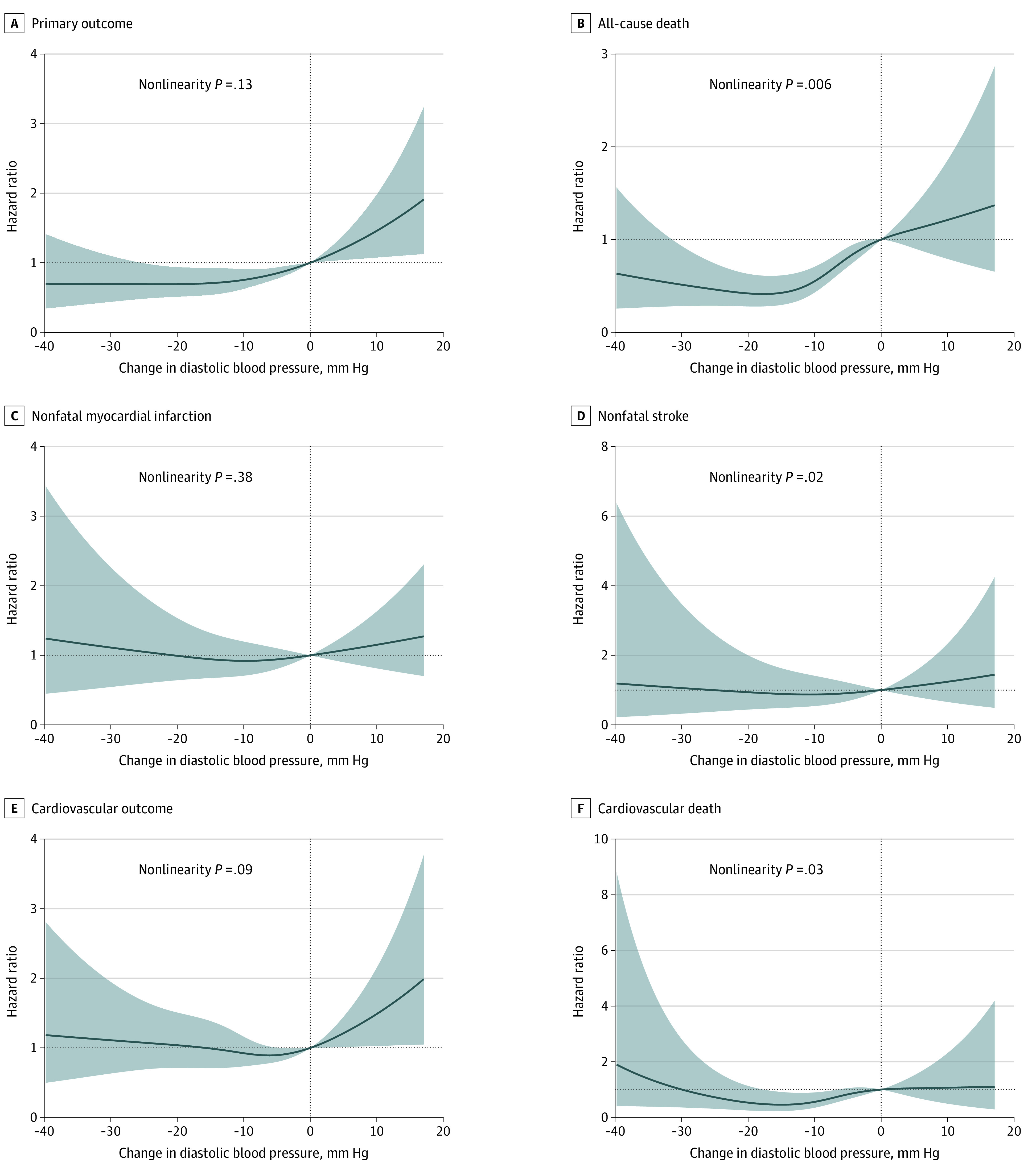
Cubic Splines for the Adjusted Hazard Ratios for Mean Diastolic Blood Pressure Change From Baseline The primary outcome consisted of all-cause death, nonfatal myocardial infarction, and nonfatal stroke. The cardiovascular outcome was composed of cardiovascular death, nonfatal myocardial infarction, and nonfatal stroke. The reference is no change in diastolic blood pressure from baseline. Shaded areas indicate 95% CIs.

To better explain the observed nonlinear association, we further analyzed treated DBP as a continuous variable using cubic spline regression adjusting for all covariates mentioned in the Methods section. Results of cubic spline regression are displayed in Figure 3, where a DBP value of 80 mm Hg was chosen as reference. Nonlinear associations between treated DBP and the primary outcome (*P* = .003), all-cause death (*P* = .001), and nonfatal myocardial infarction (*P* = .049) were observed. Similarly, nonlinear associations between DBP change from baseline as a continuous variable and all-cause death (*P* = .006), nonfatal stroke (*P* = .02) and cardiovascular death (*P* = .03) were observed (Figure 4).

## Discussion

In this study, we found that an achieved DBP value of less than 60 mm Hg was associated with significantly increased risk of the primary outcome, the composite cardiovascular outcome, nonfatal myocardial infarction, and nonfatal stroke in a population with a guideline-recommended SBP target of less than 130 mm Hg. The nominally lowest risk was observed at an achieved DBP value of between 70 and 80 mm Hg for the primary outcome, the composite cardiovascular outcome (cardiovascular death, nonfatal myocardial infarction and nonfatal stroke), nonfatal myocardial infarction, and cardiovascular death in this population.

Mainly driven by the results of SPRINT study,^16^ the 2017 AHA guidelines recommend an intensive BP target of less than 130/80 mm Hg without mentioning the lower limit of DBP.^17^ However, aggressively lowering SBP could result in a substantial reduction of DBP, counteracting the benefits of lowering SBP. Secondary analyses of SPRINT data showed that an excessively low achieved DBP was associated with a 25% increase in cardiovascular risk and even greater risk among patients with preexisting cardiovascular disease or chronic kidney disease.^25^ The linear association between DBP and outcomes reported in a previous meta-analysis was only observed at a DBP value of greater than 75 mm Hg,^5^ which might not have been low enough to observe the diastolic J-shape phenomenon. A post hoc analysis of the SPRINT study reported that even in the lowest DBP quintile (ie, <68 mm Hg), intensive BP lowering was associated with reduced risk of cardiovascular events (HR, 0.78; 95% CI, 0.57-1.07) and all-cause death (HR, 0.88; 95% CI, 0.60-1.29).^26^ However, the lowest quintile in the post hoc analysis of SPRINT may not have been low enough to reveal the potential hazardous effect, given that only 39.0% of patients achieved an treated DBP of less than 60 mm Hg in the lowest DBP quintile (ie, <68 mm Hg) in SPRINT (eTable 5 in the Supplement). When we further analyzed the association of intensive BP lowering in the subgroup with baseline DBPs of less than 60 mm Hg—a group in which more than 59.0% of patients achieved treated DBPs of less than 60 mm Hg—a nonsignificant increase in all-cause and cardiovascular death was observed in patients from both the SPRINT and ACCORD-BP trials. Although no interaction between intensive BP lowering and baseline DBP was observed, the results still forced us to reflect on whether the potential additional benefits could counteract potential risks in intensively lowering DBP to less than 60 mm Hg. What if SBP has already reached the target level? Using integrated data from SPRINT and ACCORD-BP, we found that compared with lowering DBP to 70 to 80 mm Hg, lowering DBP to less than 60 mm Hg was associated with a 46% increased cardiovascular risk in patients who achieved an SBP of less than 130 mm Hg, suggesting the existence of diastolic J-shape phenomenon even when SBP reaches the target level.

The biological plausibility for this phenomenon or the diastolic J-shape phenomenon has been proposed.^4,27,28^ Considering that ventricle perfusion occurs mostly during diastole,^29^ lower DBP could result in possible hypoperfusion and associated damage. The recently reported reversed diastolic J-shape phenomenon by myocardial reperfusion in patients with left ventricular dysfunction and heart failure after myocardial infarction^30^ and the association of low DBP with increased serum concentrations of cardiac troponin T^10^ have provided evidence for the myocardial perfusion explanation and, thus, for the diastolic J-shape phenomenon. Therefore, it is of urgent need to find safe and optimal DBP ranges for patients with treated SBPs of less than 130 mm Hg, the guideline-recommended SBP target.^17^ However, to our knowledge, no previous studies have assessed potential safe or optimal DBP targets in patients with treated SBPs of less than 130 mm Hg. Böhm et al^31^ has assessed optimal DBP in patients who achieved SBPs of 120 to 140 mm Hg, and similar to our findings, the authors found optimal DBP for these patients was between 70 and 80 mm Hg but DBP of less than 70 mm Hg (rather than 60 mm Hg) was associated with increased risk of cardiovascular events. However, many studies reporting the diastolic J-shape phenomenon^7,10,12,32^ also reported that a DBP of less than 60 mm Hg was associated with increased risk of cardiovascular events. Therefore, the present study’s findings (ie, treated DBP between 70 and 80 mm Hg was associated with the lowest outcome risk, while treated DBP of less than 60 mm Hg was associated with increased adverse outcome risk in a population with high cardiovascular risk and treated SBP of less than 130 mm Hg) emphasized the need and provided an important hypothesis for future studies aiming to explore the lower boundary of DBP targets in this population.

Reverse causality has always been a concern regarding diastolic J-shape phenomena in observational studies. Because the present study is a secondary analysis of randomized clinical trial data, reverse causality is inevitably a concern. However, we used all appropriate measures to minimize the possibility of reverse causality, including excluding patients with outcomes occurring within 30 days after randomization, adjusting age and comorbid conditions, and conducting a series of sensitivity analyses. Similar results from these sensitivity analyses implied that the findings of the present study were unlikely to be simply an effect of reverse causality. However, due to the nature of retrospective observational studies, neither this study nor previous similar studies could establish a causal relationship between low DBP and risk of outcomes. Results of the present study only serve as a caution regarding lowering DBP to less than 60 mm Hg and as an important hypothesis to be tested in future prospective studies regarding safe and optimal DBP ranges.

### Limitations and Strengths

This study has limitations and strengths. First, the present study is a post hoc analysis of previous randomized clinical trials; therefore, the possibilities of reverse causality and unidentified confounding cannot be completely ruled out, although the sensitivity analyses and adjustments we did could have mitigated these possibilities. Second, lacking biomarkers of tissue injury, such as cardiac troponin, we were unable to reveal potential mechanism of the association of low achieved DBP and cardiovascular risk. Third, there were only 476 patients (6.3%) in the subgroup with DBPs of 80 mm Hg or greater, which might lead to the observed insignificant association of DBP with all outcomes in this subgroup. Therefore, results in this DBP group should be interpreted with caution, especially the outcome of nonfatal stroke. Fourth, our findings were generated from a cohort with high cardiovascular risk; therefore, it may not apply to the general population. The strengths of present study include its large sample size, its racially diverse population, and the use of mean treated BP rather than baseline BP or BP from observational studies, which are often affected by comorbid conditions.

## Conclusions

In this study, lowering DBP to less than 60 mm Hg was associated with increased risk of cardiovascular events in patients with high cardiovascular risk and an treated SBP of less than 130 mm Hg. The finding that a DBP value between 70 and 80 mm Hg was an optimal target for this patient population merits consideration and further study.

## Notes

Supplement. **eFigure 1.** Adjusted Hazard Ratios for Mean Achieved DBP and Outcomes at Achieved SBP of Less Than 130 mm Hg in SPRINT Trial **eFigure 2.** Adjusted Hazard Ratios for Mean Achieved DBP and Outcomes at Achieved SBP of Less Than 130 mm Hg in ACCORD-BP Trial **eFigure 3.** Adjusted Hazard Ratios for Mean Achieved DBP and Outcomes at Achieved SBP of 110 to Less Than 130 mm Hg Using Integrated Data From SPRINT and ACCORD-BP **eFigure 4.** Adjusted Hazard Ratios for Mean Achieved DBP and Outcomes at Achieved SBP of Less Than 130 mm Hg Using Integrated Data From SPRINT and ACCORD-BP After Additional Adjustment of Baseline PP and Baseline DBP **eFigure 5.** Adjusted Hazard Ratios for Mean Achieved DBP and Outcomes at Achieved SBP of Less Than 130 mm Hg Using Integrated Data From SPRINT and ACCORD-BP After Additional Adjustment of Baseline PP, Baseline SBP, and Treated SBP **eTable 1.** Event Rates of the Primary and Secondary Outcomes Across Different Treated DBP Groups in Patients With Achieved SBP of Less Than 130 mm Hg **eTable 2.** Adjusted Hazard Ratios for Mean Achieved DBP and Outcomes at Achieved SBP of Less Than 130 mm Hg in ACCORD-BP Trial in Intensive and Standard Glycemic Control Group **eTable 3.** Effects of Intensive SBP Control in SPRINT on the SPRINT Primary and Secondary Outcomes in Baseline DBP of Less Than 60 mm Hg and Baseline DBP of Greater Than 60 mm Hg Subgroup **eTable 4.** Effects of Intensive SBP Control in ACCORD-BP on the ACCORD-BP Primary and Secondary Outcomes in Baseline DBP of Less Than 60 mm Hg and Baseline DBP of Greater Than 60 mm Hg Subgroup **eTable 5.** Percentage of Patients Who Achieved Treated DBP of Less Than 60 mm Hg Across Baseline DBP Quintiles in Both SPRINT and ACCORD-BP
