# Supplementary material for: Evaluation of Optimal Diastolic Blood Pressure Range Among Adults With Treated Systolic Blood Pressure Less Than 130 mm Hg
Source: JAMA Netw Open. 2021 Feb 17;4(2):e2037554. doi: 10.1001/jamanetworkopen.2020.37554 (PMC7890449; doi:10.1001/jamanetworkopen.2020.37554)
Supplement: Supplement. — eFigure 1. Adjusted Hazard Ratios for Mean Achieved DBP and Outcomes at Achieved SBP of Less Than 130 mm Hg in SPRINT Trial eFigure 2. Adjusted Hazard Ratios for Mean Achieved DBP and Outcomes at Achieved SBP of Less Than 130 mm Hg in ACCORD-BP Trial eFigure 3. Adjusted Hazard Ratios for Mean Achieved DBP and Outcomes at Achieved SBP of 110 to Less Than 130 mm Hg Using Integrated Data From SPRINT and ACCORD-BP eFigure 4. Adjusted Hazard Ratios for Mean Achieved DBP and Outcomes at Achieved SBP of Less Than 130 mm Hg Using Integrated Data From SPRINT and ACCORD-BP After Additional Adjustment of Baseline PP and Baseline DBP eFigure 5. Adjusted Hazard Ratios for Mean Achieved DBP and Outcomes at Achieved SBP of Less Than 130 mm Hg Using Integrated Data From SPRINT and ACCORD-BP After Additional Adjustment of Baseline PP, Baseline SBP, and Treated SBP eTable 1. Event Rates of the Primary and Secondary Outcomes Across Different Treated DBP Groups in Patients With Achieved SBP of Less Than 130 mm Hg eTable 2. Adjusted Hazard Ratios for Mean Achieved DBP and Outcomes at Achieved SBP of Less Than 130 mm Hg in ACCORD-BP Trial in Intensive and Standard Glycemic Control Group eTable 3. Effects of Intensive SBP Control in SPRINT on the SPRINT Primary and Secondary Outcomes in Baseline DBP of Less Than 60 mm Hg and Baseline DBP of Greater Than 60 mm Hg Subgroup eTable 4. Effects of Intensive SBP Control in ACCORD-BP on the ACCORD-BP Primary and Secondary Outcomes in Baseline DBP of Less Than 60 mm Hg and Baseline DBP of Greater Than 60 mm Hg Subgroup eTable 5. Percentage of Patients Who Achieved Treated DBP of Less Than 60 mm Hg Across Baseline DBP Quintiles in Both SPRINT and ACCORD-BP [file jamanetwopen-e2037554-s001.pdf]

Supplemental Online Content

Li J, Somers VK, Gao X, et al. Evaluation of optimal diastolic blood pressure range among adults with treated systolic blood pressure less than 130 mm Hg. *JAMA Netw Open*. 2021;4(2):e2037554. doi:10.1001/jamanetworkopen.2020.37554

eFigure 1. Adjusted Hazard Ratios for Mean Achieved DBP and Outcomes at Achieved SBP of Less Than 130 mm Hg in SPRINT Trial
eFigure 2. Adjusted Hazard Ratios for Mean Achieved DBP and Outcomes at Achieved SBP of Less Than 130 mm Hg in ACCORD-BP Trial
eFigure 3. Adjusted Hazard Ratios for Mean Achieved DBP and Outcomes at Achieved SBP of 110 to Less Than 130 mm Hg Using Integrated Data From SPRINT and ACCORD-BP
eFigure 4. Adjusted Hazard Ratios for Mean Achieved DBP and Outcomes at Achieved SBP of Less Than 130 mm Hg Using Integrated Data From SPRINT and ACCORD-BP After Additional Adjustment of Baseline PP and Baseline DBP
eFigure 5. Adjusted Hazard Ratios for Mean Achieved DBP and Outcomes at Achieved SBP of Less Than 130 mm Hg Using Integrated Data From SPRINT and ACCORD-BP After Additional Adjustment of Baseline PP, Baseline SBP, and Treated SBP
eTable 1. Event Rates of the Primary and Secondary Outcomes Across Different Treated DBP Groups in Patients With Achieved SBP of Less Than 130 mm Hg
eTable 2. Adjusted Hazard Ratios for Mean Achieved DBP and Outcomes at Achieved SBP of Less Than 130 mm Hg in ACCORD-BP Trial in Intensive and Standard Glycemic Control Group
eTable 3. Effects of Intensive SBP Control in SPRINT on the SPRINT Primary and Secondary Outcomes in Baseline DBP of Less Than 60 mm Hg and Baseline DBP of Greater Than 60 mm Hg Subgroup
eTable 4. Effects of Intensive SBP Control in ACCORD-BP on the ACCORD-BP Primary and Secondary Outcomes in Baseline DBP of Less Than 60 mm Hg and Baseline DBP of Greater Than 60 mm Hg Subgroup
eTable 5. Percentage of Patients Who Achieved Treated DBP of Less Than 60 mm Hg Across Baseline DBP Quintiles in Both SPRINT and ACCORD-BP

This supplemental material has been provided by the authors to give readers additional information about their work.

© 2021 Li J et al. *JAMA Network Open.*

eFigure 1. Adjusted Hazard Ratios for Mean Achieved DBP and Outcomes at Achieved SBP of Less Than 130 mm Hg in SPRINT Trial

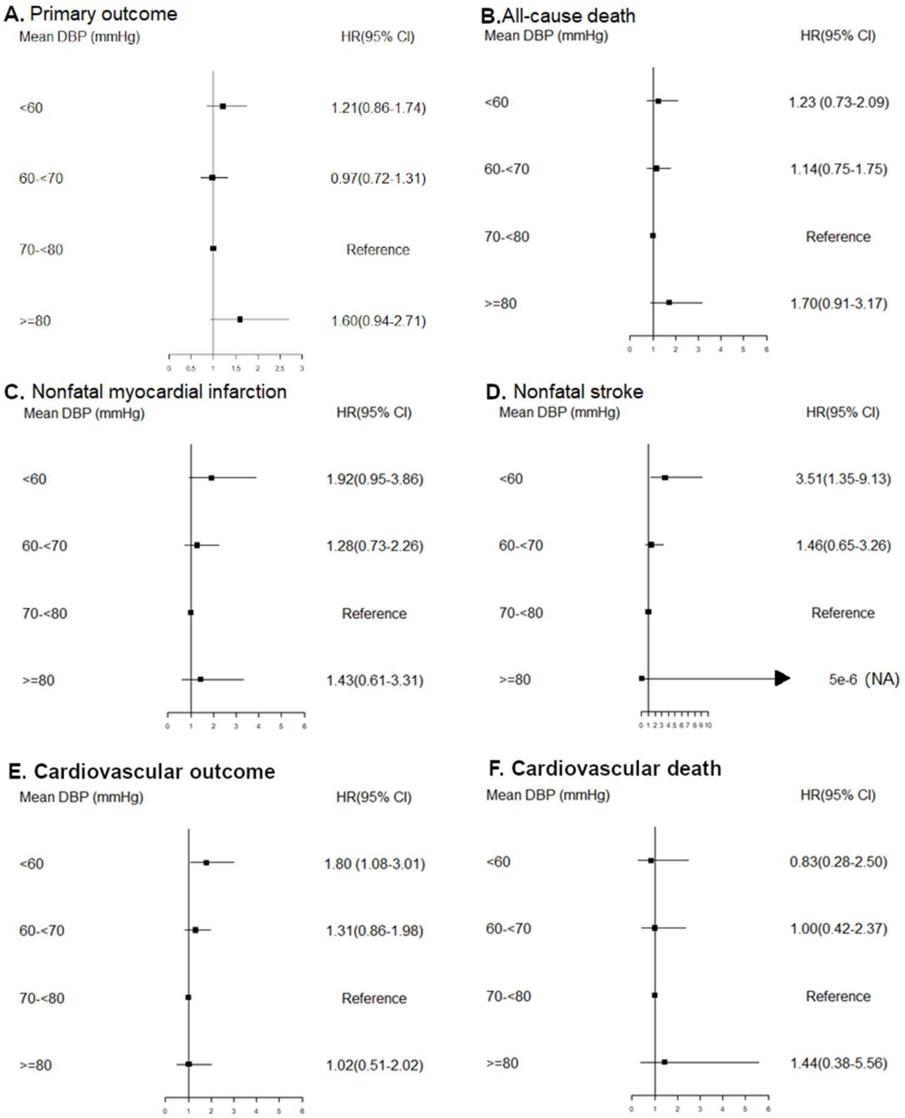

Primary outcome consisted of all-cause death, nonfatal myocardial infarction and nonfatal stroke. Cardiovascular outcome was composed of cardiovascular death, nonfatal myocardial infarction and nonfatal stroke. The reference is DBP value of 70 to <80mmHg. CI, confidence interval; DBP, diastolic blood pressure; HR, hazard ratios; SBP, systolic blood pressure.

© 2021 Li J et al. *JAMA Network Open.*

eFigure 2. Adjusted Hazard Ratios for Mean Achieved DBP and Outcomes at Achieved SBP of Less Than 130 mm Hg in ACCORD-BP Trial

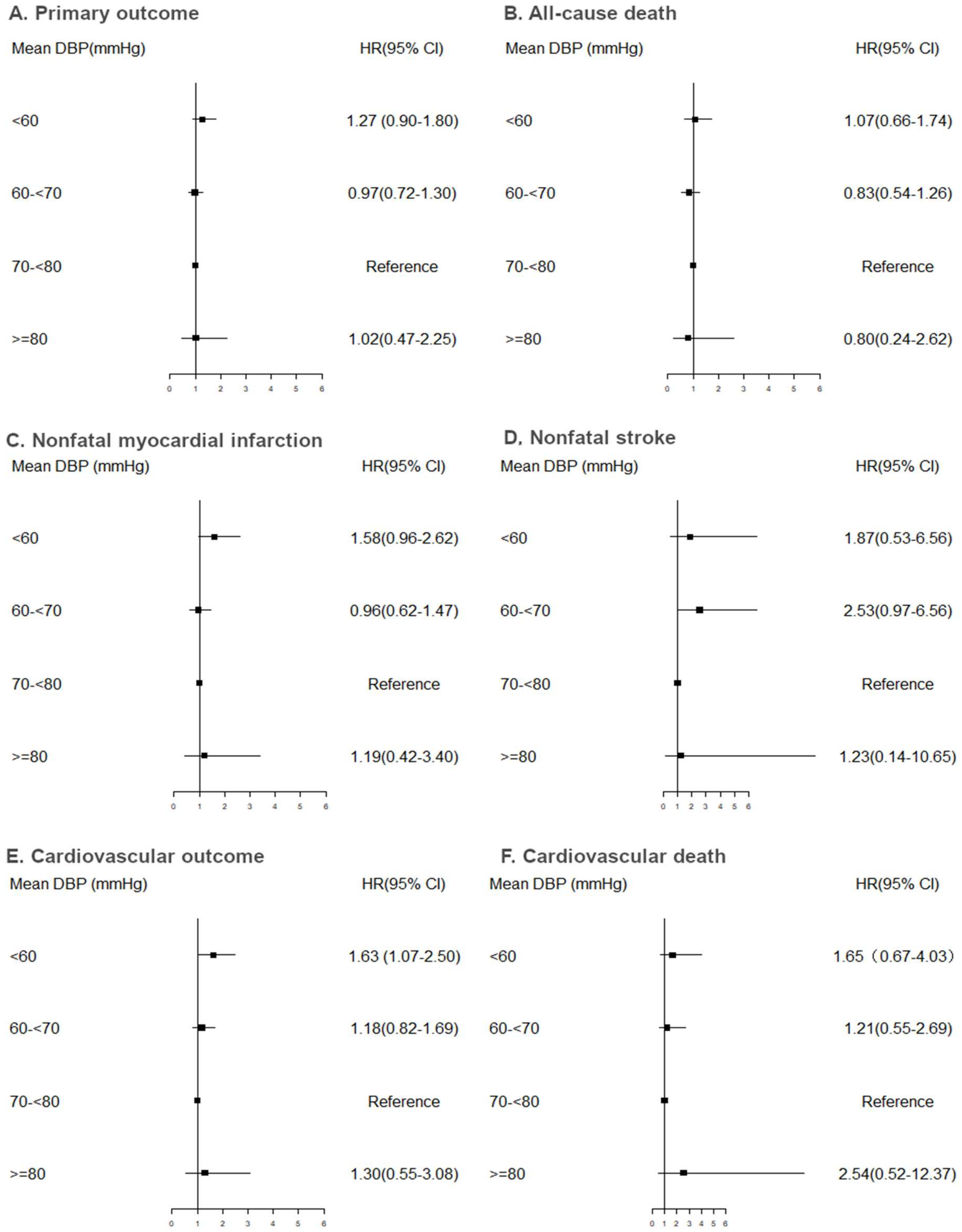

Primary outcome consisted of all-cause death, nonfatal myocardial infarction and nonfatal stroke. Cardiovascular outcome was composed of cardiovascular death, nonfatal myocardial infarction and nonfatal stroke. CI, confidence interval; DBP, diastolic blood pressure; HR, hazard ratios; SBP, systolic blood pressure.

© 2021 Li J et al. *JAMA Network Open.*

eFigure 3. Adjusted Hazard Ratios for Mean Achieved DBP and Outcomes at Achieved SBP of 110 to Less Than 130 mm Hg Using Integrated Data From SPRINT and ACCORD-BP

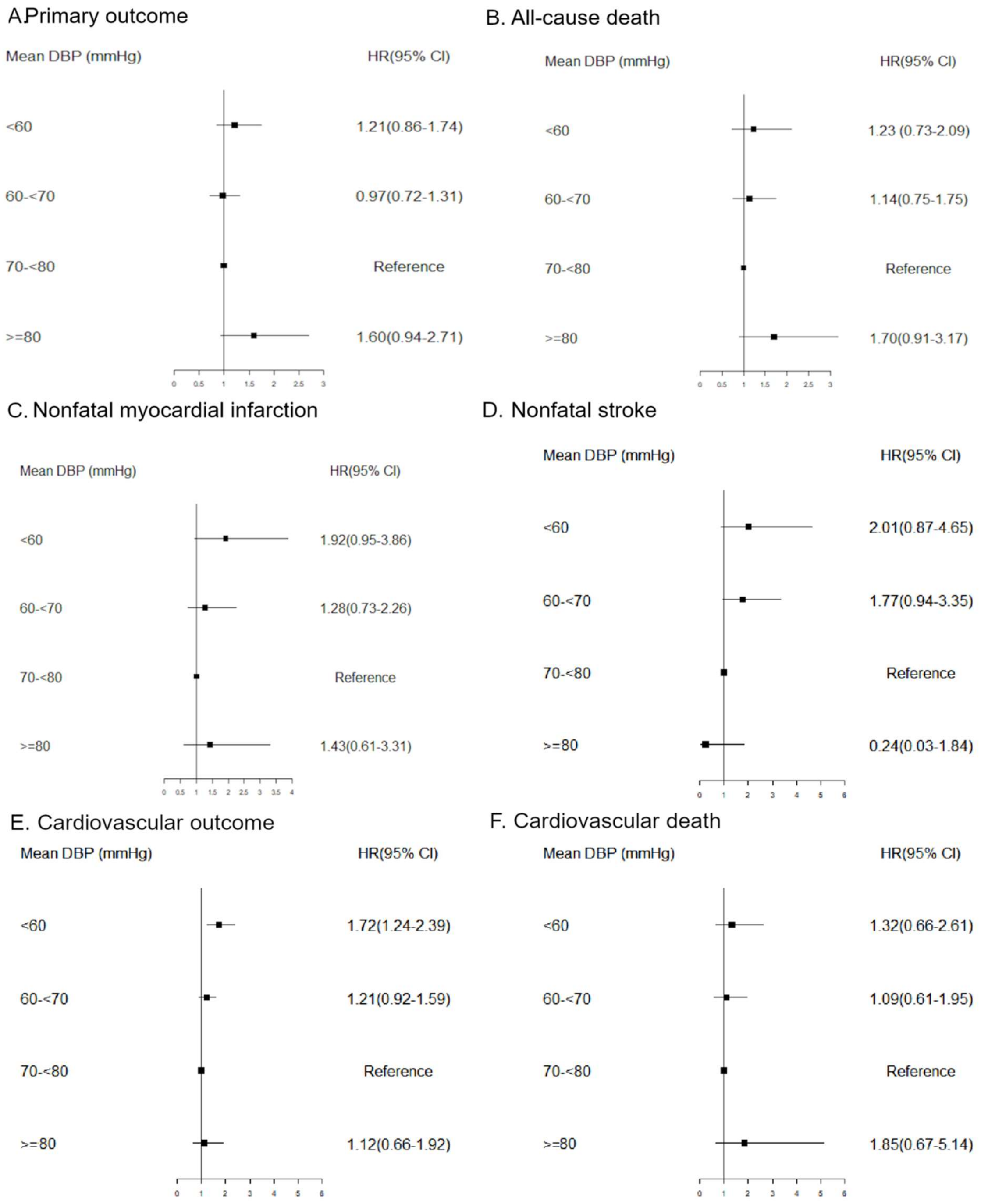

Primary outcome consisted of all-cause death, nonfatal myocardial infarction and nonfatal stroke. Cardiovascular outcome was composed of cardiovascular death, nonfatal myocardial infarction and nonfatal stroke. The reference is DBP value of 70 to <80mmHg. CI, confidence interval; DBP, diastolic blood pressure; HR, hazard ratios; SBP, systolic blood pressure.

© 2021 Li J et al. *JAMA Network Open.*

eFigure 4. Adjusted Hazard Ratios for Mean Achieved DBP and Outcomes at Achieved SBP of Less Than 130 mm Hg Using Integrated Data From SPRINT and ACCORD-BP After Additional Adjustment of Baseline PP and Baseline DBP

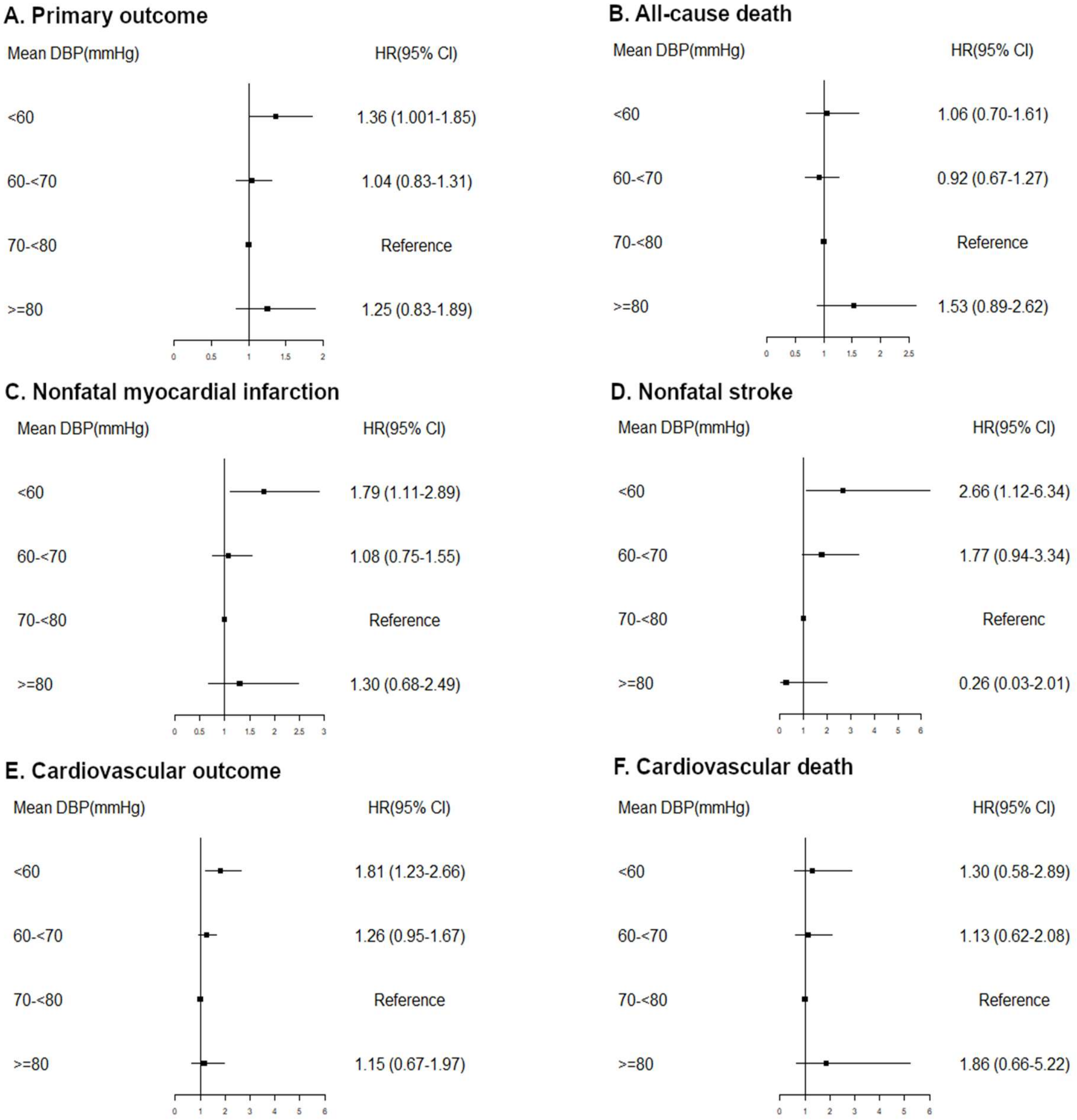

Primary outcome consisted of all-cause death, nonfatal myocardial infarction and nonfatal stroke. Cardiovascular outcome was composed of cardiovascular death, nonfatal myocardial infarction and nonfatal stroke. The reference is DBP value of 70 to <80mmHg. CI, confidence interval; DBP, diastolic blood pressure; HR, hazard ratios; PP, pulse pressure; SBP, systolic blood pressure.

© 2021 Li J et al. *JAMA Network Open.*

eFigure 5. Adjusted Hazard Ratios for Mean Achieved DBP and Outcomes at Achieved SBP of Less Than 130 mm Hg Using Integrated Data From SPRINT and ACCORD-BP After Additional Adjustment of Baseline PP, Baseline SBP, and Treated SBP

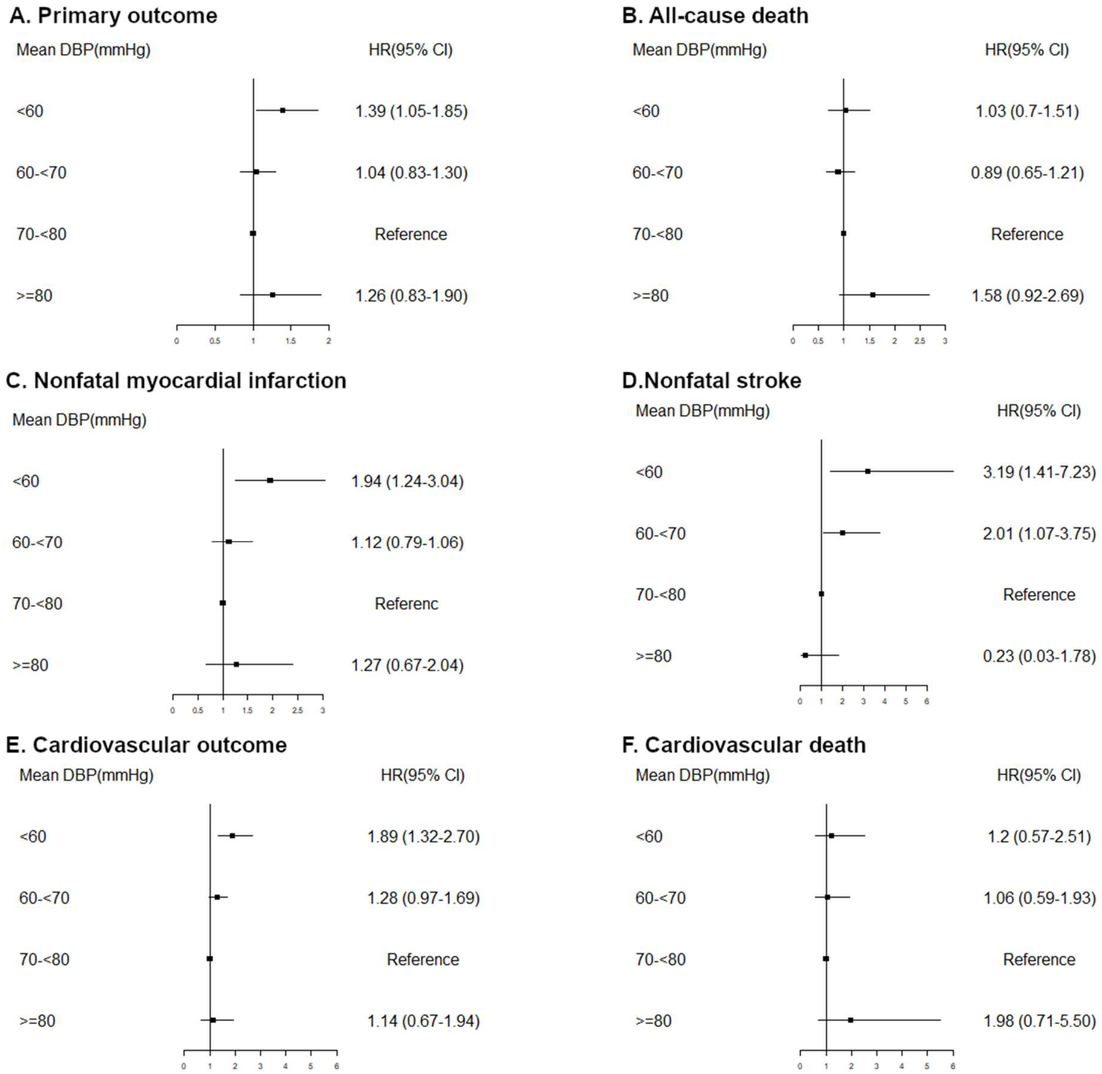

Primary outcome consisted of all-cause death, nonfatal myocardial infarction and nonfatal stroke. Cardiovascular outcome was composed of cardiovascular death, nonfatal myocardial infarction and nonfatal stroke. The reference is DBP value of 70 to <80mmHg. CI, confidence interval; DBP, diastolic blood pressure; HR, hazard ratios; PP, pulse pressure; SBP, systolic blood pressure.

© 2021 Li J et al. *JAMA Network Open.*

eTable 1. Event Rates of the Primary and Secondary Outcomes Across Different Treated DBP Groups in Patients With Achieved SBP of Less Than 130 mm Hg

DBP groups / Outcomes	<60mmHg (n=1244)	60-<70mmHg (n=3250)	70-<80mmHg (n=2545)	≥80mmHg (n=476)
Primary outcome*	168 (13.5)	261 (8.0)	149(5.9)	29 (6.1)
All-cause death	92 (7.4)	138 (4.2)	77(3.0)	18 (3.8)
Nonfatal myocardial infarction	72 (5.8)	100 (3.1)	61 (2.4)	12 (2.5)
Nonfatal stroke	20 (1.6)	38 (1.2)	18 (0.7)	1 (0.2)
Cardiovascular outcome#	111 (8.9)	175 (5.4)	92 (3.6)	17 (3.6)
Cardiovascular death	28 (2.3)	41 (1.3)	19 (0.7)	5 (1.1)

* Primary outcome was composed of all-cause death, nonfatal myocardial infarction and nonfatal stroke

# Cardiovascular outcome was composed of cardiovascular death, nonfatal myocardial infarction and nonfatal stroke.

DBP, diastolic blood pressure; SBP, systolic blood pressure.

© 2021 Li J et al. *JAMA Network Open.*

eTable 2. Adjusted Hazard Ratios for Mean Achieved DBP and Outcomes at Achieved SBP of Less Than 130 mm Hg in ACCORD-BP Trial in Intensive and Standard Glycemic Control Group

Outcomes	Mean achieved DBP (mmHg)	Intensive glycemic control group, HR (95% CI)	Standard glycemic control group, HR (95% CI)
Primary outcome*	< 60	1.18 (0.72, 1.96)	1.38 (0.85, 2.24)
	60-<70	0.91 (0.59, 1.39)	1.02 (0.67, 1.53)
	70-<80	reference	Reference
	≥80	1.14 (0.40, 3.28)	0.99 (0.30, 3.25)
All-cause death	< 60	1.43 (0.70, 2.92)	0.81(0.41, 1.60)
	60-<70	1.07 (0.57, 2.03)	0.64 (0.36, 1.15)
	70-<80	Reference	Reference
	≥80	1.23 (0.27, 5.56)	0.54 (0.07, 4.08)
Nonfatal myocardial infarction	< 60	0.94 (0.45, 1.98)	2.51 (1.23, 5.11)
	60-<70	0.66 (0.36, 1.22)	1.31 (0.71, 2.45)
	70-<80	Reference	Reference
	≥80	1.04 (0.23, 4.68)	1.50 (0.34, 6.68)
Nonfatal stroke	< 60	1.24 (0.10, 15.13)	2.32 (0.43, 12.44)
	60-<70	4.45 (1.01, 19.61)	2.52 (0.61, 10.32)
	70-<80	Reference	Reference
	≥80	5.03 (0.42, 59.97)	-
Cardiovascular outcome#	< 60	1.30 (0.70, 2.41)	2.03 (1.12, 3.67)
	60-<70	1.04 (0.62, 1.72)	1.34 (0.80, 2.23)
	70-<80	Reference	Reference
	≥80	1.64 (0.55, 4.91)	1.03 (0.24, 4.46)
Cardiovascular death	< 60	2.90 (0.72, 11.65)	0.86 (0.24, 3.08)
	60-<70	2.02 (0.55, 7.39)	0.71 (0.25, 2.05)
	70-<80	Reference	Reference
	≥80	7.13 (1.08, 47.23)	-

* Primary outcome was composed of all-cause death, nonfatal myocardial infarction and nonfatal stroke

# Cardiovascular outcome was composed of cardiovascular death, nonfatal myocardial infarction and nonfatal stroke.

CI, confidence interval; DBP, diastolic blood pressure; HR, hazard ratios; SBP, systolic blood pressure.

© 2021 Li J et al. *JAMA Network Open.*

eTable 3. Effects of Intensive SBP Control in SPRINT on the SPRINT Primary and Secondary Outcomes in Baseline DBP of Less Than 60 mm Hg and Baseline DBP of Greater Than 60 mm Hg Subgroup

	**Baseline DBP <60 mmHg (n=538)**	**Baseline DBP ≥60 mmHg (n=8823)**	***P* for interaction**
Primary outcome*			0.784
No.(%) of events	52 (9.7)	510 (5.8)	
HR (95% CI)	0.96 (0.56, 1.66)	0.74 (0.62, 0.88)	
All-cause death			0.290
No.(%) of events	36 (6.7)	329 (3.7)	
HR (95% CI)	1.25 (0.65, 2.34)	0.70 (0.56, 0.87)	
Cardiovascular death			0.200
No.(%) of events	5(0.9)	97 (1.1)	
HR (95% CI)	1.69 (0.28, 10.12)	0.53 (0.35, 0.81)	
Average on-treatment DBP (mmHg), mean (SD)	58.8(7.7)	72.4 (8.8)	-
On-treatment DBP <60 mmHg, n (%)	316 (59.5)	694(8.0)	-

* SPRINT primary outcome, composed of myocardial infarction, other acute coronary syndromes, stroke, heart failure, or death from cardiovascular causes.

CI, confidence interval; DBP, diastolic blood pressure; HR, hazard ratios; SD, standard deviation.

© 2021 Li J et al. *JAMA Network Open.*

eTable 4. Effects of Intensive SBP Control in ACCORD-BP on the ACCORD-BP Primary and Secondary Outcomes in Baseline DBP of Less Than 60 mm Hg and Baseline DBP of Greater Than 60 mm Hg Subgroup

	**Baseline DBP <60 mmHg (n=261)**	**Baseline DBP ≥60 mmHg (n= 4470)**	***P* for interaction**
Primary outcome*			0.688
No.(%) of events	45 (17.2)	400 (8.9)	
HR (95% CI)	0.78 (0.43, 1.41)	0.91 (0.74, 1.10)	
All-cause death			0.226
No.(%) of events	36 (13.8)	258 (5.8)	
HR (95% CI)	2.00 (1.001, 4.00)	1.00 (0.78, 1.27)	
Cardiovascular death			0.175
No.(%) of events	18 (6.9)	100 (2.2)	
HR (95% CI)	1.25 (0.49, 3.16)	1.04 (0.70, 1.54)	
Average on-treatment DBP(mmHg), mean (SD)	58.91 (6.37)	69.29 (7.68)	-
On-treatment DBP <60 mmHg, n (%)	155 (59.8)	513 (11.6)	-

* ACCORD-BP primary outcome, composed of nonfatal myocardial infarction, nonfatal stroke, or death from cardiovascular causes.

CI, confidence interval; DBP, diastolic blood pressure; HR, hazard ratios; SD, standard deviation.

© 2021 Li J et al. *JAMA Network Open.*

eTable 5. Percentage of Patients Who Achieved Treated DBP of Less Than 60 mm Hg Across Baseline DBP Quintiles in Both SPRINT and ACCORD-BP

<table>
<tr><th></th><th>Trial</th><th>Quintile 1</th><th>Quintile 2</th><th>Quintile 3</th><th>Quintile 4</th><th>Quintile 5</th></tr>
<tr><td rowspan="4">Achieved DBP <60 mmHg</td><td rowspan="2">SPRINT</td><td><68 mmHg</td><td>68-74 mmHg</td><td>75-80 mmHg</td><td>81-87 mm Hg</td><td>≥88 mm Hg</td></tr>
<tr><td>672 (39.0)</td><td>222 (11.9)</td><td>78(4.4)</td><td>30(1.6)</td><td>8(0.4)</td></tr>
<tr><td rowspan="2">ACCORD-BP</td><td><67 mmHg</td><td>67-73 mmHg</td><td>73-78 mmHg</td><td>78-85 mmHg</td><td>>85 mmHg</td></tr>
<tr><td>385 (44.7)</td><td>150 (16.6)</td><td>67 (7.5)</td><td>51(4.6)</td><td>15 (1.6)</td></tr>
</table>

DBP, diastolic blood pressure

© 2021 Li J et al. *JAMA Network Open.*
